# Filarids (Spirurida: Onchocercidae) in wild carnivores and domestic dogs from the Brazilian Atlantic forest

**DOI:** 10.1371/journal.pntd.0010213

**Published:** 2022-03-08

**Authors:** Marcela Figuerêdo Duarte Moraes, Andressa de Souza Pollo, Estevam Guilherme Lux Hoppe

**Affiliations:** São Paulo State University–Unesp, Agrarian and Veterinarian Sciences School (FCAV), Department of Pathology, Reproduction, and One Health, Jaboticabal, Brazil; University of Agricultural Sciences and Veterinary Medicine Cluj-Napoca, Life Science Institute, ROMANIA

## Abstract

Onchocercidae nematodes are heteroxenous parasites with worldwide distribution, and some of the species associated to animals may present zoonotic potential. Climatic changes and anthropic influences on the environment may result in vectors’ proliferation, facilitating the spillover to humans and/or non-typical animal hosts. The Iguaçu National Park (PARNA Iguaçu), one of the most important Brazilian natural remanescents of Atlantic rainforest, is strongly affected by human activities such as tourism and agriculture. The complexity of this area is especially characterized by the close nexus between the rich wildlife, humans, and domestic animals, especially domestic dogs. Based on this, this research aimed to diagnose the Onchocercidae nematodes in wild carnivores and domestic dogs in the PARNA Iguaçu and the surrounding areas. For this, we collected 162 samples of seven species of wild carnivores and 225 samples of domestic dogs. The presence of microfilariae in the blood samples was diagnosed by the modified Knott’s test and molecular screening, and the specific identification was based on sequencing of the *myoHC* and *hsp70* genes. Microfilariae were detected only in ring-tailed coatis, in which we found five species: *Mansonella* sp. 1, *Mansonela* sp. 2, Onchocercidade gen. sp. 1, Onchocercidade gen. sp. 2, and *Dirofilaria immitis*. The morphological analysis supported the molecular findings. The domestic dogs were parasitized by *Acanthocheilonema reconditum*, representing a new locality record for this species. Phylogenetic analysis showed high genetic similarity among the four undetermined species and *Mansonella* spp., *Brugia* spp., and *Wuchereria bancrofti*. The presence of *D*. *immitis* in ring-tailed coatis may be result of spillover from dogs, even though the parasite was not diagnosed in the sampled dogs. The presence of several undetermined Onchocercidae species indicates the necessity of continuous investigations on wild and domestic animals from Neotropical area, especially considering the growing anthropic influence on forest remnants.

## Author summary

Filarids are vector-borne Onchocercidae nematodes widely distributed around the world, related to several hosts, both wild and domestic, in different Biomes, including human-related species. In consequence to deforestation, environmental degradation, and climate changes, there has been an expansion in the distribution of vector-borne diseases around the world, such as those caused by filarids, in endemic tropical areas and even in temperate regions where they did not used to occur before. Thus, epidemiology and molecular genetic diversity studies of these filarial parasites are of great importance for three main reasons: zoonotic potential of many known species of filarids; the lack of data about the species inhabiting neotropical regions and the current worldwide deforestation and consequent habitat loss scenario, which favors the emergence or re-emergence of diseases resulting from the infection of domestic animals and humans by parasites that used to have as reservoirs only wild animals.

## 1. Introduction

The Onchocercidae nematodes are represented by 88 genera and constitute an important group of vector-borne spirurids with wordwide distribution [1,2]. The adult forms of filarids can be found in blood or lymphatic vessels, heart, lungs, body cavities, articulations, and subcutaneous tissues, while their larvae, named microfilariae, are located mainly in blood or subcutaneous tissues. The severity of the disease caused by these nematodes is related to the parasite species, the location of the adult form and to the lesions caused by the microfilariae in the tissues. Nonetheless, most part of Onchocercidae nematodes are apathogenic [3].

The World Health Organization included three diseases caused by Onchocercidae nematodes, namely river blindness, lymphatic filariosis, and dracunculiosis, are included in the Tropical Neglected Diseases list [4]. The most studied filariosis are those that affect humans, as the Bancroftian filariosis, caused by *Wuchereria brancofti*; the river blindness, caused by *Onchocerca volvulus*, the African and Asian lymphatic filarioses, caused by *Brugia timori*, *Brugia pahangi*, *Brugia malayi*, and *Loa loa* [5]; and mansonellosis caused by *Mansonella streptocerca* in Africa, *Mansonella perstans* in African and American continents, and *Mansonella ozzardi*, commonly found in Latin America and endemic in the Brazilian Amazonia [6–9].

However, other filarids genera that parasitize wild and/or domestic animals have also been related to human infection, especially species of the *Dirofilaria*, *Onchocerca*, *Brugia*, *Dipetalonema*, and *Acanthocheilonema* genera. These filarids were originally described affecting animals, and only later their zoonotic potential was revealed [10]. *Dirofilaria repens* and *Dirofilaria immitis* are the most well documented zoonotic filarids [11], but other species are known to infect humans [12]. As other vector-borne parasites, Onchocercidae nematodes may be favored by climate changes and anthropic actions, as these factors are related to the rise of vector populations and their dispersion to new areas [13].

The richness of the Onchocercidae species in Brazil is remarkable, with 26 genera comprising 91 species related to several hosts, from amphibians to humans [14–16]. However, until 2008, only four *Dirofilaria* species were known to affect wild carnivores: *Dirofilaria incrassata*, *Dirofilaria striata*, *Dirofilaria repens*, and *Dirofilaria spectans* [14–15,17–19]. Later, in 2017, three other species, *Dirofilaria immitis*, *Mansonella* sp., and *Brugia* sp., were diagnosed in wild ring-tailed coatis from Brazil, based on morphological analyses of their microfilariae [16].

The Iguaçu National Park (PARNA Iguaçu) is one of the most important Brazilian Atlantic rainforest remnants, with high biological diversity of fauna and flora. Despite the conservation efforts, the PARNA Iguaçu is highly affected by anthropic actions in its border areas, especially due to grain monocultures and livestock activity, presence of roads and highways, and irregular human settlements [20]. Furthermore, the PARNA Iguaçu has strong touristic activities, receiving more than one million tourists from all over the world per year [21]. Currently, many small and fragmented populations of wild carnivores persist in this Park. Their conservation status and close relation to humans and domestic animals result in vulnerability to infectious diseases, including those caused by pathogens with a wide geographic and host distribution and which, in turn, can also pose risk to human health [22–24].

Health monitoring of wild animals may contribute to evaluate possible circulation of zoonotic pathogens, especially in areas prone to disease spillover, with close nexus between wildlife, domestic animals, and humans [25]. Generalist and abundant animal species may act as good sentinels, and then, monitoring the health tatus of wild carnivores of the park, as well as the domestic dogs living close to the area may be useful for disease surveillance [26]. For this purpose, this research aimed to diagnose filarioses in wild carnivores and domestic dogs in the PARNA Iguaçu and the surrounding areas.

## 2. Material and methods

### 2.1 Ethical aspects

All the procedures adopted in this research are in accord to international standarrds and were approved by the local Ethics Committee on Animal Use of São Paulo State University—Unesp, Agrarian and Veterinarian Sciences School—FCAV (Protocol 07553/14) and by the Brazilian Authorization and Information System—SISBIO (Protocol 38006–2).

### 2.2 Study area and animal trapping

**Fig 1:**
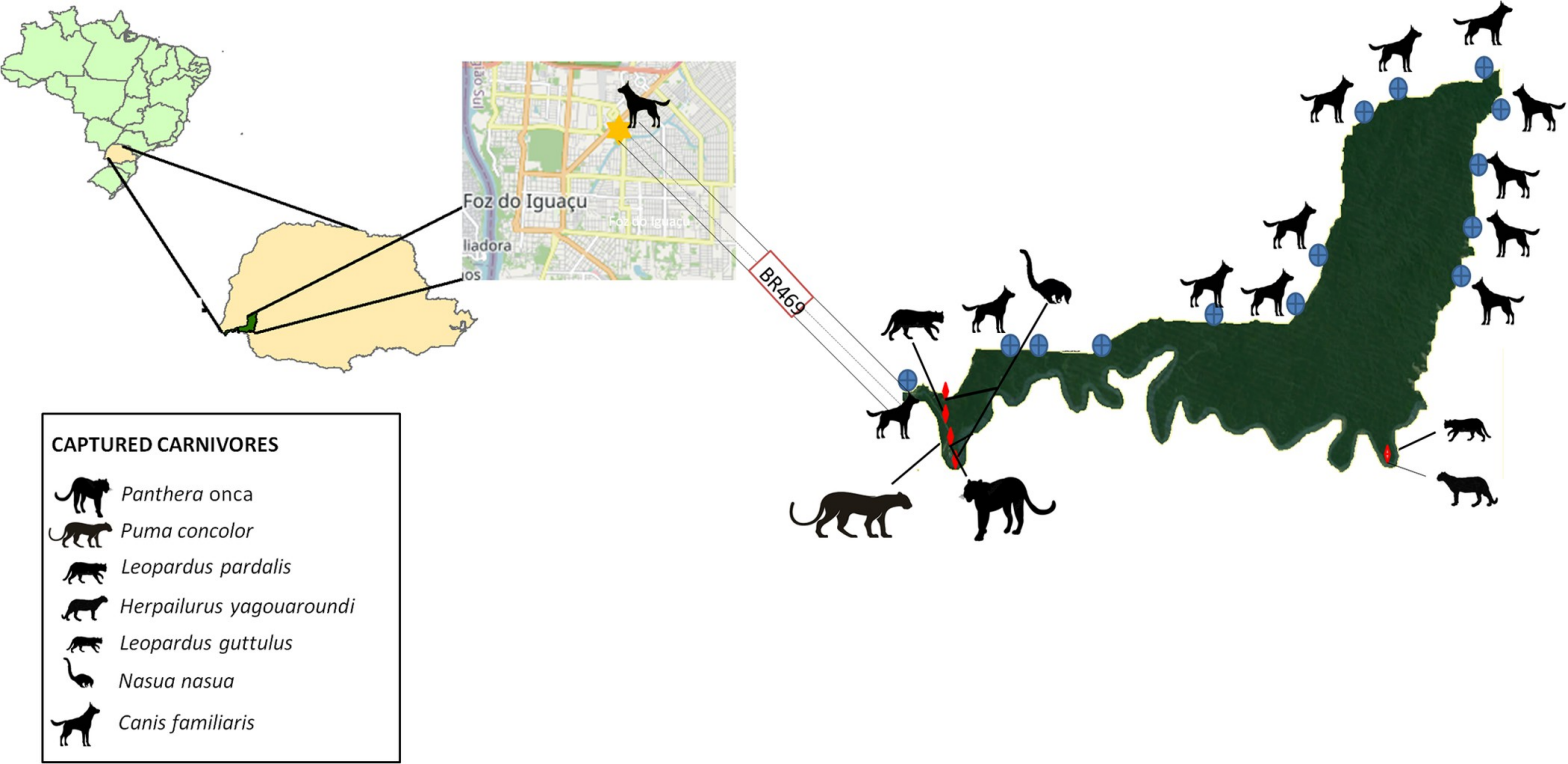
Geographic location of Iguaçu National Park, Brazil, points of capture and number of wild carnivores and domestic dogs sampling. (Red diamonds represent the sites of captures of wild carnivores; Blue circles represent the sites of domestic dogs samplings in rural areas; Yellow star represents the site of domestic dogs sampling in urban area) Source of base layer https://www.openstreetmap.org/#map=9/-25.6613/-54.7833.

PARNA Iguaçu is located within the Atlantic Forest Biome in the western state of Paraná, Brazil, representing one of the last remants of this important Biome (25^o^05’S to 25^o^40’S and 54^o^30’W to 54^o^40’W) [27] (Fig 1). The area has mesothermal humid climate, with anual rainfall ranging from 1500 to 2000 mm [27]. The Central and Southern parts of PARNA Iguaçu have submontane semideciduous seasonal forest vegetation, and the Northern area is characterized as mixed ombrophilous forest vegetation. The fauna of the Park is highly diverse, with registers of 102 mammals, 386 birds, 48 reptiles, 13 amphibians, 176 fishes, 839 invertebrates, and 28 species of parasite helminths, most of them endemic to the Atlantic rainforest [28].

The small to medium-sized wild carnivores were captured with hand nets and Tomahawk traps baited with a mixture of fresh chicken meat, fruits (banana, pineapple or mango) and peanut butter, and the large carnivores were trapped in snares. All the field procedures were in several campaigns, developed from from August 2014 to December 2017. Following the captures, the animals were physically restrained, anesthetized with tiletamine-zolazepan combination (5mg/Kg, IM), and identified with a small numbered eartag to prevent recaptures. The blood samples were drawn from the jugular vein and then stored in plain and EDTA-coated tubes in isothermal boxes with ice. In addition, we collected samples of liver and spleen tissue of 23 recently roadkilled wild carnivores found on the BR-469 road, located inside the PARNA Iguaçu (Table 1).

**Table 1:** Source of the studied wild carnivores and type of collected sample from PARNA Iguaçu.

Animal	Trapped specimens (n)	Roakilled carcasses (n)	Sampled tissues (n)	Total specimens (n)
Crab-eating foxes *Cerdocyon thous*	0	4	S/L (4)	4
Ring-tailed coatis *Nasua nasua*	135	17	B (135), S/L (17)	152
Ocelots *Leopardus pardalis*	1	0	B (1)	1
Southern tiger cat *Leopardus guttulus*	1	2	B (1), S/L (2)	3
Jaguarundi *Herpailurus yagouaroundi*	1	0	B (1)	1
Jaguar *Panthera onca*	1	0	B (1)	1
Cougar *Puma concolor*	1	0	B (1)	1
**Total**	**140**	**23**	**B (140), S/L (23)**	**163**

N number, B blood, S/L spleen and liver fragments

Regarding the domestic dogs, we sampled 225 animals from 64 rural settlements from eight different cities around the PARNA Iguaçu, and from one dog shelter at the urban area of Foz do Iguaçu, the most populated city in this region. All the sampled areas, including the dog shelter, are located within a range of 1 Km from the PARNA Iguaçu borders. For sample collection, the dogs were physically restrained under the supervision of their owners and the blood samples were drawn from the cephalic vein in plain and EDTA-coated tubes and placed in isothermal boxes with ice.

### 2.3. Microfilariae detection

Microfilariae detection in the blood was performed according to the Knott’s technique, modified by Newton and Wright [29]. For the morphological analysis, we obtained images of at least 100 microfilariae of each blood sample, in an Olympus BX-51 microscope equipped with a QColor 3 digital camera, processing the morphometric data with ImagePro Plus v. 4.0 software.

### 2.4 Molecular studies

#### 2.4.1 DNA extraction and molecular screening

DNA extraction of all blood and tissue samples was developed following methodology proposed by Bag et al. [30]. Cellular lysis of 250 μL of blood of each animal or 0.2g of tissue, occurred in 750 μL of DNA extraction buffer (160 mM Tris-HCl; 60 mM EDTA; 20 mM NaCl; 1% SDS; 1% PVP40; 0.2% β-mercaptoethanol) with glass beads treated in Triton-X solution 1%, at 65°C for 40 min. Proteins were precipited by adding 250 μL of potassium acetate 5M and ice bathing for 20 min. After centrifugation at 12000 x g for 10 min at 4°C, the supernatant was transferred to a new microtube and submitted to two cleaning steps with 500 μL of chloroform:isoamyl alcohol (24:1) at same centrifugation conditions previously mentioned. The clear supernatant was then transferred to a microtube containing 1000 μL of absolute ethanol to enable DNA precipitation at -20°C for 12 h. After centrifugation at 12000 x g for 20 min, the aqueous phase was discarded, and the obtained pellet was washed in 1000 μL of 70% ethanol by centrifugation at the same conditions mentioned before. Finally, the pellet was dried at 55°C and resuspended in 50 μL of TE solution (Tris-HCl 10 mM; EDTA 1 mM).

The microfilaremia screening was performed by applying the set of primers S2/S16 [31]. The reactions were composed by 1X buffer (50 mM KCl, 200 mM TRIS-HCl, pH 8.4); 2 mM MgCl_2_; 0.2 mM dNTP’s; 0.5 U Platinum Taq DNA polymerase (Invitrogen); 5 pmol of primers; BSA 0.001 mg, 100 ng of total genomic DNA, and nuclease free water to 20 μL. The amplification occurred on a Nexus thermocycler (Eppendorf) programmed to perform one cycle at 95°C for 3 minutes; 35 cycles at 94°C for 30 seconds, 56°C for 30 seconds, and 72°C for 40 seconds; and a final cycle at 72°C for 10 minutes. Samples with negative results for microfilariae even after reamplification of the PCR product were submitted to another PCR to detect the presence of GAPDH mammalian gene [32], in order to verify the integrity of the sample and absence of PCR-inhibiting substances.

#### 2.4.2 Molecular identification and phylogeny

For this study, we designed primers for amplification of the *myoHC* and *hsp70* genes under single nucleotide polymorphisms (SNPs) regions, focusing on the genera previously identified in this area [16], based on the sequences of seven genomic regions of Onchocercidae nematodes [2] and *Wuchereria bancrofti* genome draft [33] (Table 2). The quality and stability of these newly designed primer sequences were evaluated with the software Primer3Plus [34].

**Table 2:** Primers designed for amplification of *myoHC* and *hsp70* genes of selected Onchocercidae species.

Genes	Filarid Genera	Primer name*	Primer sequence (5’– 3’)	T°C	bp size
*myoHC*		MyDiros-F	AGAAACTGAAGCCCAAGCAA		
	*Dirofilaria* spp.	MyDiroimmitis-R	TGGTTTGCAGTTCTGCGATTT	50°C	535
		MyDirorepens_R	AGACGGGTTTTCAAGGTGGT	52°C	590
	*Brugia* spp. Sheathed species	MyBru-F	TGGAAGAAATTGAAAGACAGAGACA		
		MyBru-R	GTTAAGCCGTGTCTTTAGTGTCG	55°C	646
		MyWu-R	CCGTGTCTTTAATGTCGTAAATTC	52°C	640
	*Mansonella* spp. *Dipetalonema* spp.	MyMan-F	GAAGCTGAGGCTCAAGCAAT		
		MyMan-R	TCTGTTTTGCTCATCGCATT	52°C	554
		MyDipet-R	AGCCGTGTCTTTAATGTTGTGA	52°C	700
*hsp70*		h70Diro-F	ATCCCGACGAAAACRTCTCA		
	*Dirofilaria* spp.	h70Diroimmitis-R	CTGCAATGCGATCTTTCTGCGC	56°C	445
		h70Dirorepens-R	TGCTGCGATACGATCTTTCTG	52°C	435
	*Brugia* spp. Sheathed species	h70Bru-F	TGCACTCATCAAGAGAAATACCA	52°C	483
		h70Bru-R	GCAATACGATCCTTCTGTGCT		
	*Mansonella* spp.	h70Man-F	TGAGACAGCTGGAGGTGTTATG	55°C	484
		h70Man-R	ATCTTTCTGTGCCTCATCATCTG		

* The F primers can be combined with each correspondent R primers. The annealing temperatures and amplicon size refer to each pair matching

All samples tested positive for Onchocercidae nematodes were submitted to *myoHC* and *hsp70* genes amplification using each set of primers designed, following the same protocol described for microfilaremia screening, followed by a reamplification with the same set of primers. The amplicons were sequenced with the BigDye Terminator v3.1 kit (Applied Biosystems), according to the manufacturer’s instruction. Sequencing was performed in an ABI3130 sequencer (Applied Biosystems).

The consensus sequences were obtained and trimmed considering phred bases quality higher than 20, using the Phred/Phrap/Consed package [35–37]. Sequences of each gene were aligned individually with sequences from the NCBI database using the MUSCLE tool [38]. The best evolutionary model for each alignment was obtained according to the Akaike Information Criterion (AIC) with the software ModelTest 3.7 [39]. Bayesian phylogenetic trees were obtained using the evolutionary models GTR and HKY+I for the *myoHC* and *hsp70* genes, respectively, both with gamma distribution, using the MrBayes 3.2.3 software [40]. Two MCMC runs were performed concomitantly using ten million generations with four chains, and the trees were sampled every 100 generations. At the end of the analyses, with standard deviation lower than 0.01, 25% of the initial generated trees were discarded as burn-in. The phylogenetic trees were generated with all sequences obtained, although, in order to facilitate the visualization, the final edition of the trees contains only the haplotypes selected with the DnaSP6 software [41]. The phylograms were graphically edited with Dendroscope 3 software [42]. The nematodes *Protospirura muricola* and *Filaria latala* were used as outgroups (Access numbers KP760231, KP760257, KP760429, and KP760455).

#### 2.4.4 Immunological test for Dirofilaria immitis antigen

The Dirofilariasis AG Kit immunochromatographic test (ALERE, United States of America) was used as complement for *D*. *immitis* diagnosis in animals tested positive for this parasite in molecular tests. The results were processed and interpreted according to the manufacturer’s recommendations.

## 3. Results

### 3.1 Knott’s test screening and morphology

We obtained blood samples from 140 wild carnivores, mainly from the Southeastern area of the PARNA Iguaçu, where the touristic activities are concentrated. Regarding the domestic dogs, we sampled 225 animals, 140 from rural settlements close to the PARNA Iguaçu border, four stray dogs captured in Tomahawk traps inside the Park area, and 81animals from the dog shelter at the urban area of Foz do Iguaçu.

Microfilariae were detected by the Knott’s test in 84.4% (114/135) of the ring-tailed coatis and in 8% (18/225) of the domestic dogs. We found four different morphospecies in the ring-tailed coatis, while the domestic dogs were affected by a single species: *Acanthocheilonema reconditum*.

The ring-tailed coatis had two slender, unsheated microfilariae, morphologically compatible with the *Mansonella* genus, named *Mansonella* sp. 1 and *Mansonella* sp. 2. The other two morphospecies observed in these procyonids were sheated microfilariae, named Onchocercidae gen. sp. 1 and Onchocercidae gen. sp. 2.

**Fig 2:**
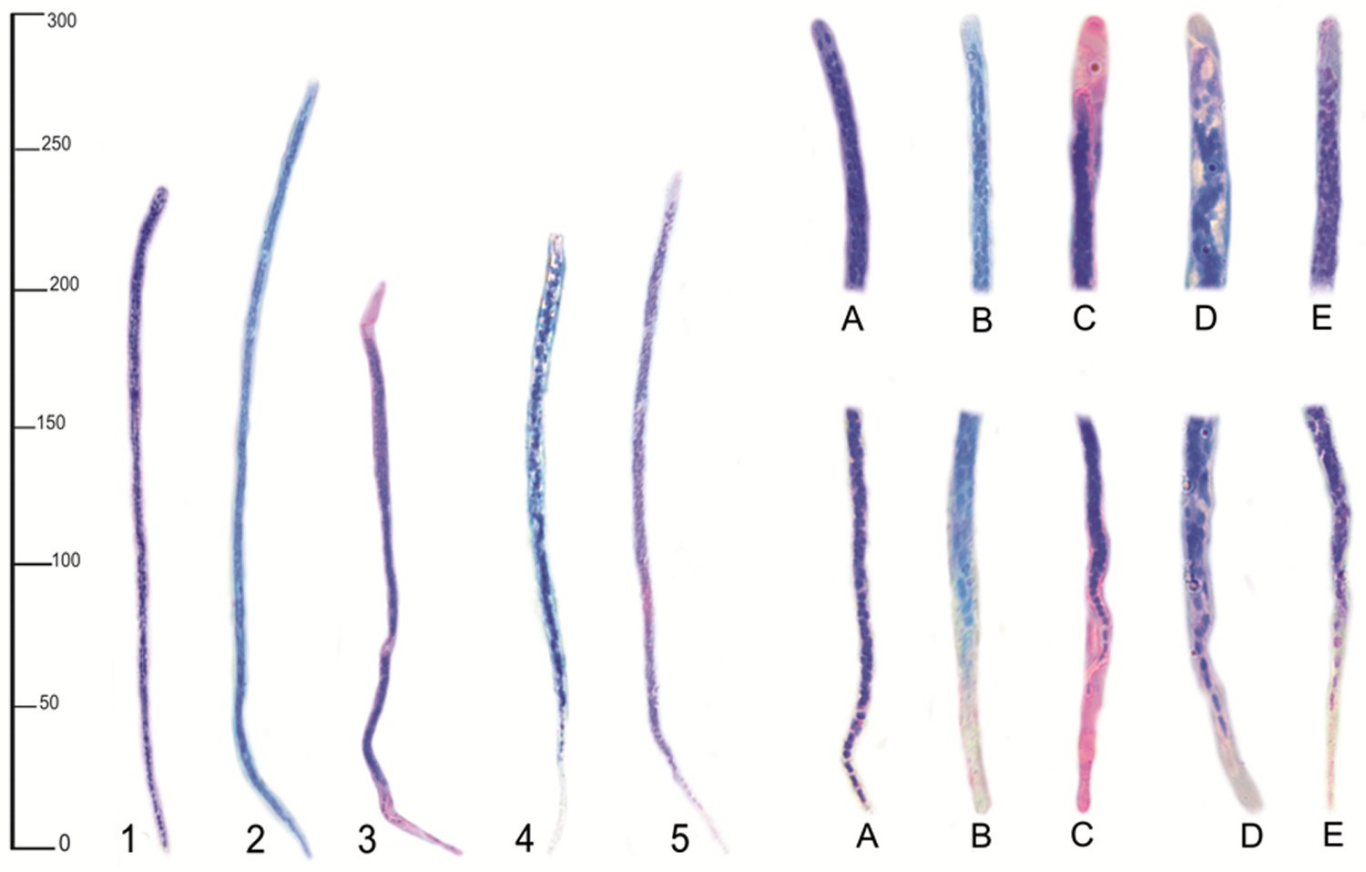
Morphology of the microfilariae detected in ring-tailed coatis (*Nasua nasua*) and domestic dogs from the studied area. 1. *Mansonella* sp. 1: A- Anterior and posterior; 2. *Mansonella* sp. 2: B—Anterior and posterior; 3. Onchocercidae gen. sp. 1—C: anterior and posterior; 4. Onchocercidae gen. sp. 2—D: Anterior and posterior; 5. *Acanthocheilonema reconditum*—E: anterior and posterior. 100x magnification light microscopy.

*Mansonella* sp. 1 are slender and unsheathed microfilariae, with overall length of 233.4 ± 27.2 μm, 4.05 μm wide. The cephalic space has two well-defined amphids and cephalic hook is absent. The body is completely filled with numerous cell bodies, with well-defined anatomical structures. The nerve ring is located at 27.1 ± 4.6 μm, and excretory pore at 47 ± 4.3 μm from the anterior ending, respectively. The anal pore opens at 46.2 ± 7.7 μm from the tail tip. The tail gradually tapers into a hooked tip, with small coalesced cell bodies arranged in a single row (Figs 2 and 1A).

The other putative species of this genus, *Mansonella* sp. 2, has unsheathed microfilariae, with the cephalic space fully filled with cell bodies, also without cephalic hook. The body is 276 ± 13.1 μm long and 5.4 ± 1.7 μm wide, with coalesced cell bodies and well-defined anatomical structures. Nerve ring and excretory pore are situated at 55.3 ± 16.3 μm 85.4 ± 11.1 μm from the anterior ending. The anal pore is located at 56.7 ± 4.1 μm from the tail, which is lacking cell bodies and tapers abruptly at its end (Fig 2B).

**Fig 3:**
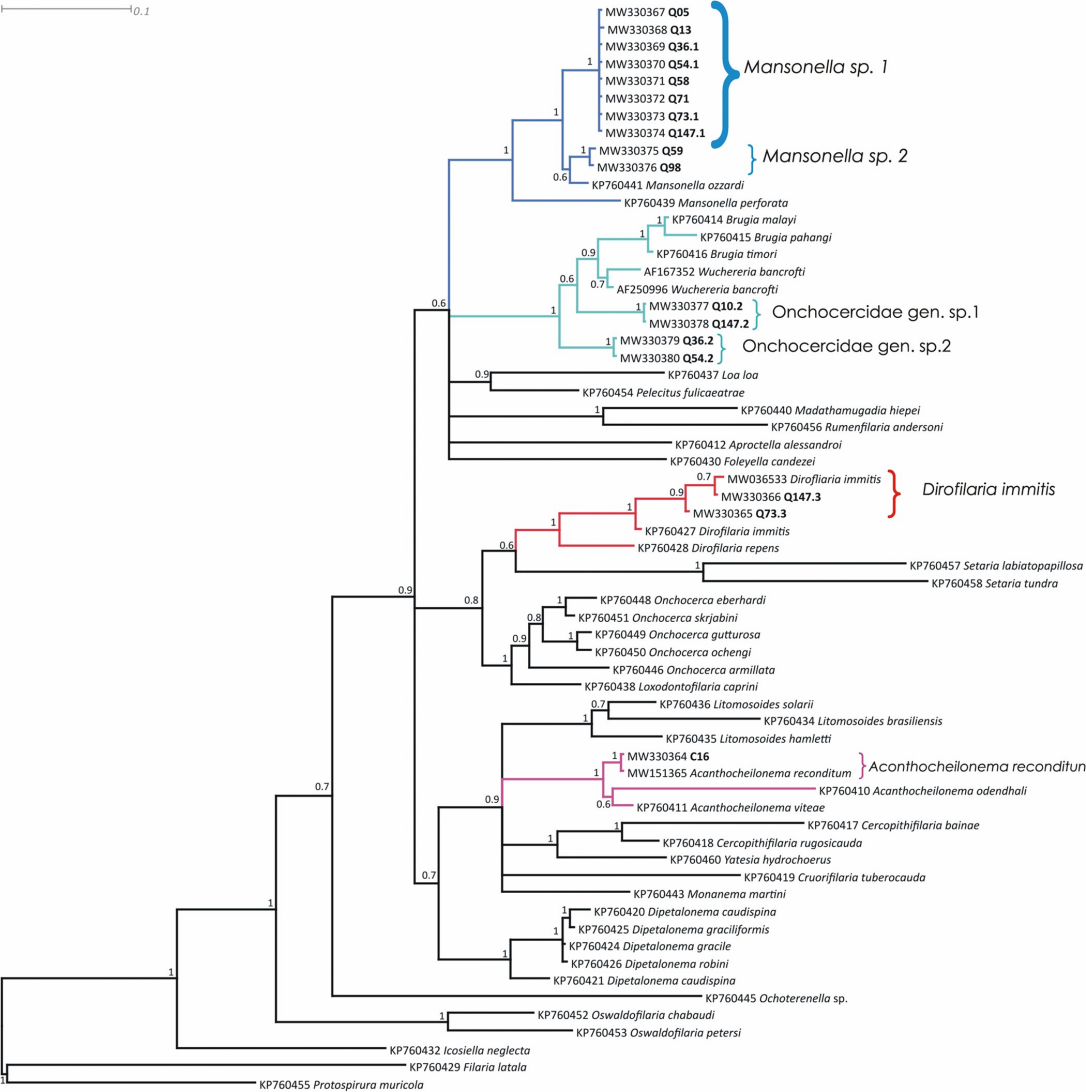
Bayesian phylogenetic tree of *myoHC* gene sequences of Onchocercidae microfilariae haplotypes present in blood samples of ring-tailed coatis (*Nasua nasua*) and domestic dog from the Iguaçu National Park. Isolates from this study are highlighted. The blue branches are related to *Mansonella* spp clade, the cyan branches are related to the sheated species clade, the red branches represent *Dirofilaria immitis* clade, and the magenta branches represent *Acanthocheilonema reconditum*.

The Onchocercidae gen. sp. 1 microfilariae are sheated, with a shorter anterior sheath measuring 13.7 ± 2.5 μm, and a longer, elongated, conical-shaped posterior sheath with 28.7 ± 4.1 μm in length. The cephalic hook is absent. The body measures 208.8 ± 12.8 μm, 6.02 ± 0.48μm in width, and it is completely filled with small coalesced cell bodies. The nerve ring is located at 31.3 ± 2.2 μm and the excretory pore at 71.5 ± 1.8 μm from the anterior ending. The tail of these microfilariae is tapered and has eight cell bodies at its anterior portion, followed by an anucleated space, ending with a single cell body. The anal pore is located at 38.9 ± 1.9 μm from the tail ending (Figs 2 and 3C).

**Fig 4:**
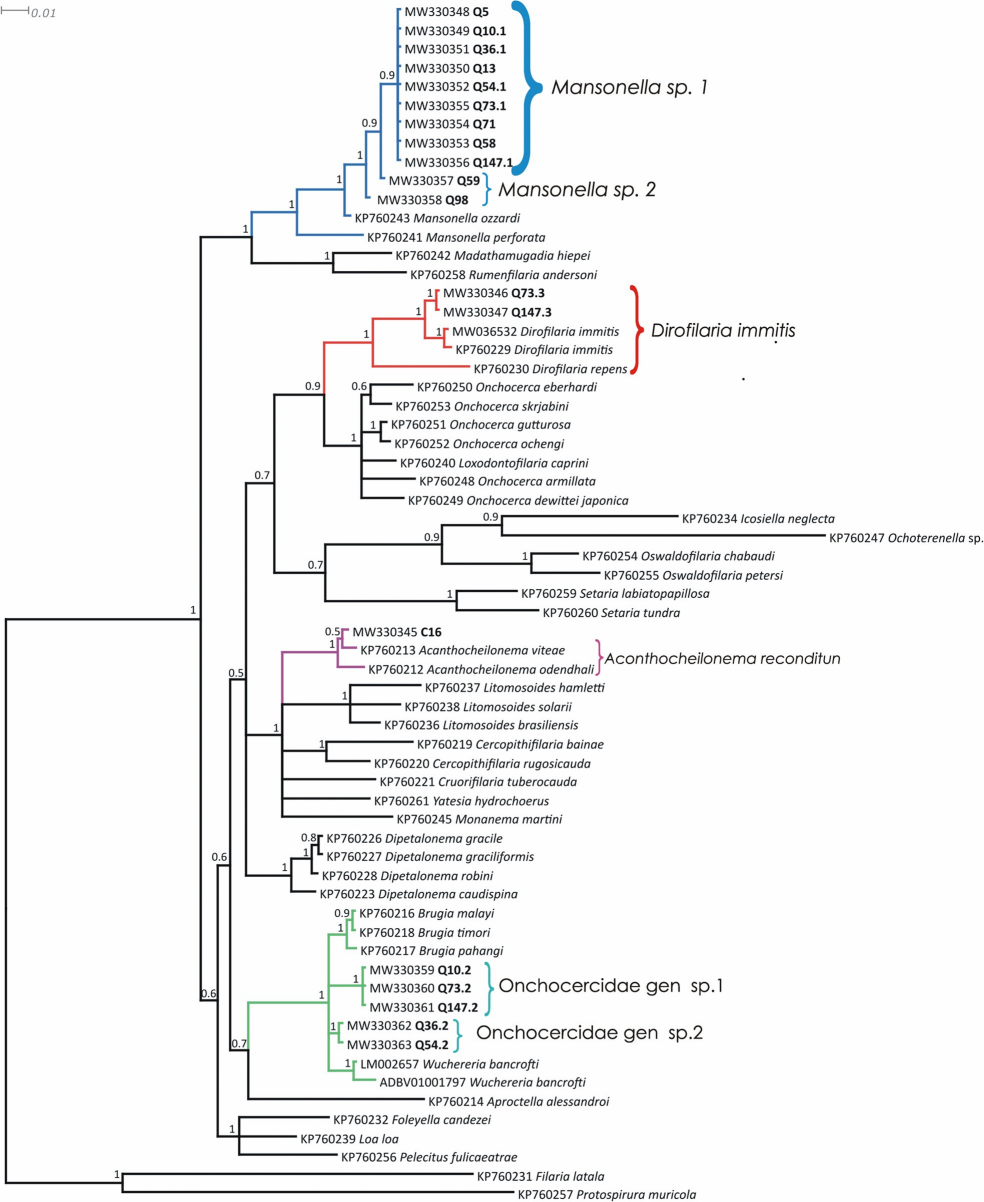
Bayesian phylogenetic tree of *hsp70* gene sequences of Onchocercidae microfilariae haplotypes present in blood samples of ring-tailed coatis (*Nasua nasua*) and domestic dog from the Iguaçu National Park. Isolates from this study are highlighted. The blue branches are related to *Mansonella* spp clade, the red branches represent *Dirofilaria immitis* clade, the magenta branches represent *Acanthocheilonema reconditum*, and the the green branches are related to the sheated species clade.

The microfilariae of the last putative species diagnosed in the ring-tailed coatis, Onchocercidae gen. sp. 2, is characterized by the presence of a cephalic hook and short sheath. These microfilariae measure 216.4 ± 6.5 μm in total length and 6.08 ± 0.5 μm in width. The anterior part of the sheath measures 5.5 ± 4.1 μm in length, and the posterior part is 11.2 ± 3.7 μm long. Cell bodies are absent in the cephalic space, but they are numerous in the rest of the microfilariae body. In the initial third of these microfilariae, the cell bodies are smaller and spaced, which giving a mottled pattern to this portion of the body. Nerve ring was not visualized in any of the specimens, and the excretory pore is situated at 43.9 ± 4.2 μm from the anterior ending. The tail has eight lined up cell bodies, with a clear gently tapered tail tip. The anal pore opens at 38.1 ± 2.8 μm from the tail tip (Figs 2 and 4D).

**Fig 5:**
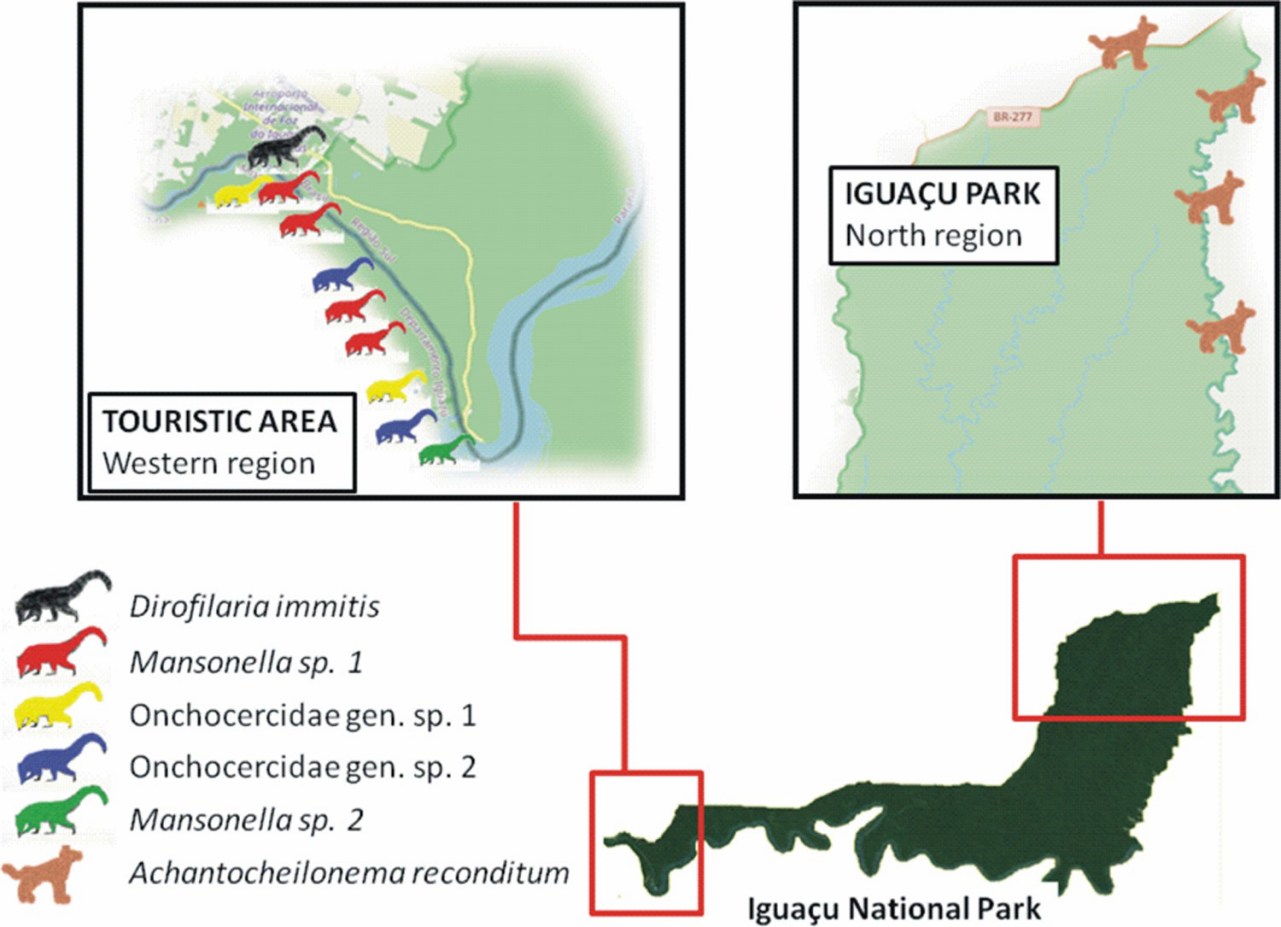
Spatial distribution of the ring-tailed coatis (*Nasua nasua*) and domestic dogs infected by Onchocercidae nematodes, PARNA Iguaçu. Source of base layer https://www.openstreetmap.org/#map=9/-25.6613/-54.7833.

The domestic dogs were affected only by *Acanthocheilonema reconditum*, characterized as unsheathed microfilariae measuring 242.1 ± 7.5 μm in length and 4.67 ± 0.37 μm wide. Cell bodies are absent in the cephalic space, but the rest of the microfilariae body is filled with numerous coalesced small cell bodies. The nerve ring and excretory pore are located at 25.5 ± 3 μm and 74.5 ± 5.2 μm from the anterior ending, respectively. The tail has five lined up cell bodies, with a clear terminal part. The anal pore opens at 52.2 ± 12.6 μm from the tail ending and the tail ends into a pointed tip (Figs 2 and 5E).

### 3.2 Immunochromatographic rapid test for Dirofilaria immitis antigen

The samples from the ring-tailed coatis identified as Q73 and Q147 tested positive for *D*. *immitis* antigen. We used as control test other eight blood samples identified as Q19, Q22, Q25, Q28, Q41, Q49, Q59 and Q60, all positive to microfilariae other than *D*. *immitis* in Knott’s and molecular tests. The test evaluated in this study did not showed cross-reaction to other species.

### 3.3 Molecular tests and phylogenetic analyses

Both blood and tissue samples were screened for microfilariae. We obtained positive results for 65.08% (102/155) of the ring-tailed coatis and 1.77% (04/225) of the domestic dogs. All the other animals were negative on screening reactions, even though they tested positive for gapdh, indicating the presence of viable DNA and absence of PCR inhibitors. The designed primers (Table 2) allowed the amplification of unique bands that, when sequenced, corresponded to a single target species.

*Mansonella* was the most prevalent genus in the ring-tailed coatis, being detected in 98.04% (100/102) of the positive samples to filarids according to the molecular screening. The clade correspondent to *Mansonella* species was composed by two haplotypes: *Mansonella* sp. 1, represented by the samples Q5, Q10.1, Q13, Q36.1, Q54.1, Q58, Q71, Q73.1, and Q147.1; and *Mansonella* sp. 2, detected on samples Q59 and Q98 (Figs 3 and 4). *Mansonella* sp. 1 was the most prevalent species, found in 98 of the sequenced samples. This putative species showed a genetic identity of 98.5% and 92.6% for the *myoHC* and *hsp70* genes of *Mansonella ozzardi*, respectively. *Mansonella* sp. 2 presented 98.5% (Q59) - 99% (Q98) of identity with *M*. *ozzardi* for *myoHC* gene, both showing 96.3% of identity with *hsp70* gene sequence of the same species. Notably, the *myoHC* gene sequences obtained from the two positive samples presented 99.4% when compared to each other.

Animals Q10, Q36, Q54, Q73, and Q147 were coinfected with *Mansonella* sp. 1 and the sheated microfilariae Onchocercidae gen. sp. 1 and Onchocercidae gen. sp. 2. The phylogenetic analyses of both *myoHC* and *hsp70* genes placed the sheated microfilariae in the same clade of *Brugia pahangi*, *Brugia malayi*, *Brugia timori*, and *Wuchereria bancrofti*. Onchocercidae gen. sp. 1 and sp. 2 presented genetic identity of 97.7% and 90.4% with *Brugia* spp. and *Wuchereria bancrofti*, respectively, for both *myoHC* and *hsp70* genes. Based on morphology and phylogenetic data, we could infer that these putative new species are associated to lymphatic vessels.

Two ring-tailed coatis, Q73 and Q147 were co-infected by three species, *Mansonella* sp. 1, Onchocercidae gen. sp. 1, and *Dirofilaria immitis*. The sequences Q73.3 and Q147.3 of both *myoHC* and *hsp70* genes are identical to each other and presented an average of 98.2% of identity with *D*. *immitis* sequences of the GenBank.

Regarding the domestic dogs, only the sample C16 amplified with the primer sets MyMan-F/MyBru-R and Hman-F/HBru-R, revealing infection by *Acanthocheilonema reconditum* according to the *hsp70* phylogenetic analysis. The C16 *hsp70* sequence presented 99.8% of identity with *A*. *reconditum* and 97% of identity with *A*. *vitae* sequences from the GenBank. *MyoHC* could not be compared due to lack of data on this databank.

### 3.4 Overall results ans spacial distribution

The results of the different techniques for diagnostic of onchocercid infection in wild carnivores and domestic dogs in PARNA Iguaçu are described in Table 3. The ring-tailed coatis infected with *Dirofilaria immitis* were captured close to the borders, while the other species had similar distribution in the border and central areas of the PARNA Iguaçu, especially in the touristic area. On the other hand, domestic dogs infected with *Acanthocheilonema reconditum* were concentrated in the border area close to the Northern portion Park (Fig 5).

**Table 3:** Prevalence of Onchocercidae nematodes in ring-tailed coatis and domestic dogs from the Iguaçu National Park, in relation to the diagnostic tests.

	Domestic Dogs	Ring-Tailed Coatis
**Modified Knott test (MK)** ***Mansonella sp*** Onchocercidae gen. sp. 1 Onchocercidae gen. sp. 2 *Dirofilaria immitis* *Acanthocheilonema reconditum*	7.9% (18/225) ND* ND ND ND 10.6% (24/225)	84.4% (114/135) 78,51% (106/135) 13.33% (18/135) 2.2% (3/135) 1.48% (2/135) ND
**PCR screening**	1.7% (4/225)	75.5% (102/135)
**Sequencing**	25% (1/4)	98.04% (100/102)
*Mansonella sp*. 1	ND	98.04% (100/102)
*Mansonella sp*. 2	ND	1.96% (2/102)
Onchocercidae gen. sp. 1	ND	2.94% (3/102)
Onchocercidae gen. sp. 2	ND	1.96% (2/102)
*Dirofilaria immitis*	ND	1.96% (2/102)
*Acanthocheilonema reconditum*	25% (1/4)	ND
**Immunochromatographic test for *Dirofilaria immitis***	ND	1.96% (2/102)

*ND not detected

## 4. Discussion

The molecular and morphological analysis of the blood samples of domestic dogs and carnivores associated to the PARNA Iguaçu, showed the occurrence of five Onchocercidae species representing three distinct genera in the ring-tailed coatis, while the domestic dogs presented infection by only one species. In Brazil, only *D*. *immitis* and *Mansonella* spp. had been reported in ring-tailed coatis [7,14,16]. Considering domestic dogs, there are a few reports regarding *A*. *reconditum*, but none in the studied area, indicating the lack of data on Neotropical Onchocercidae. The tissue samples did not amplify in the screening tests, even though there was viable DNA in the samples, and these results could be related to absence of microfilariae rather than technique failure. The samples of the carnivores other than ring-tailed coatis also tested negative, certainly due to the low sample size.

In temperate regions, there are several Onchocercidae species associated with carnovores, such as *Dirofilaria immitis*, *D*. *repens*, *D*. *ursi*, *Dipetalonema arbuti*, *Onchocerca lupi*, *Acanthocheilonema reconditum*, and *Acanthocheilonema dracunculoides* [43,44]. However, considering the the Neotropical region, there are little information on the diversity of these nematodes, despite the greater biodiversity and variety of biomes in this ecozone. Evolutionarily, the long isolation of South America, followed by the Great American Interchange, in Cenozoic Era, had great importance for the South American filarid diversity, once this continent has spent most of this geological Period isolated from the other continents [45]. During this geological Period occurred the main Onchocercidae diversification, as a consequence of the appearance of birds and mammals and adaptative species radiation, which created new host niches for this group of parasites [46].

In South America, the *Dipetalonema* lineage is the most ancient Onchocercidae. This lineage was first reported in marsupials and xenartrhans, subsequently adapting to caviomorph rodents and platyrrhine monkeys [47]. The influx of mammals to South America after the Panama isthmus formation resulted in the introduction of *Brugia*, *Lithomosoides*, and *Cercopithifilaria* in South America, contributing to the complex biodiversity of this area [46]. The comprehension of the Onchocercidae introduction and diversification process in the Neotropical region is essential to explain the high species richness observed in this study. This research was the first to perform a phylogenetic study of Neotropical wild carnivore filarids, but previous data indicated the presence of *Mansonella*, *Dirofilaria*, and *Brugia* in ring-tailed coatis in the PARNA Iguaçu, based on morphological analysis [16]. For this reason, molecular analysis using conserved genes that allowed to group the species more accurately, in association with morphological analysis in the present study, constituted an essential approach to reach the results obtained. The primers designed for the target genes had good performance, preventing mixing of amplicons with similar sizes related with different species and non-specific amplifications, unlike reactions targeting the ribosomal 18S and ITS regions [48–51].

Phylogenetically, the Onchocercidae nematodes can be divided into five distinct clades. The Clade 1 is composed by basal species associated to amphibians and reptiles, Clade 2 is composed by the Setariinae subfamily, the Clade 3 is formed by the basal species of the Dirofilariinae and Onchocercinae subfamilies, the Clade 4 consists of the *Dipetalonema* lineage, and the Clade 5 is formed by the most derived species of the Dirofilariinae, Onchocercinae, and Splendidofilariinae subfamilies, including those associated to human infections [2]. The studied ring-tailed coatis were parasitized by species of the Clades 3 and 5. *Acanthocheilonema reconditum*, diagnosed in the domestic dogs, is classified in Clade 4. The undetermined species associated to ring-tailed coatis *Mansonella* sp. 1, *Mansonella* sp. 2, Onchocercidae gen. sp. 1, and Onchocercidae gen. sp. 2 are related to Clade 5, suggesting that species associated with this procyonid carnivore are primarily constituted of newer and derived species.

*Mansonella* was the predominant genus identified, found in most of the ring-tailed coatis sequenced samples. The genus *Mansonella* is one of the most diverse Onchocercidae genera, with species related to carnivores, sciurids, thupaids, ungulates, and primates, including humans, mainly in Africa and South America [1]. The *Mansonella* genus is divided into seven subgenera: *Mansonella* (*Mansonella*), *Mansonella* (*Tupainema)*, *Mansonella* (*Tetrapetalonema)*, *Mansonella* (*Esslingeria*), *Mansonella* (*Cutifilaria*), *Mansonella* (*Filyamagutia*), and *Mansonella* (*Pseudolitomosa*) [52]. The *Mansonella* (*Mansonella*) subgenus is a parasite of Holartic carnivores and sciurids, and also of humans in South America. *Mansonella* (*Tupainema*) is a monospecific subgenus, related only to Tupaiidae species, Order Scandentia. *Mansonella* (*Tetrapetalonema*) is associated to platyrrine monkeys from South America. The subgenus *Mansonella* (*Esslingeria*) is found in anthropoid primates and humans in Africa, and South America, and in Caviomorpha rodents from South America. *Mansonella* (*Cutifilaria*) was described in deer in Europe and Asia. The last two subgenera, *Mansonella* (*Filaymagutia*) and *Mansonella* (*Pseudolitomosa*) were described in Japan, the former in Ursidae species and the latter in Sciuridae rodents [53].

Our results revelaed the presence of two putative species, *Mansonella* sp. 1 and *Mansonella* sp. 2. The classification of the *Mansonella* subgenera is based on the morphology of the microfilariae, particularly the arrangement of the cell bodies in the tail [54]. *Mansonella* sp. 1 has cell bodies all along the microfilaria tail, including the tail tip, similar to the observed in *Mansonella* (*Cutifilaria*), *Mansonella* (*Esslingeria*), and *Mansonella* (*Tetrapetalonema*). As the classification in one of these subgenera depends on adult characteristics, we could not suggest any further., but none of the subgenera traditionally associated to carnivores have this type of cell bodies distribution on the microfilariae. Regarding *Mansonella* sp. 2, the microfilaria morphology is similar to that of *Mansonella* (*Mansonella*) *ozzardi* [54]. Considering this, in addition to the close phylogenetic relationship observed for these taxa in phylogenetic analyses, this undetermined species can be classified in the same subgenus.

The other two putative species, Onchocercidae gen. sp. 1 and Onchocercidae gen. sp. 2, show a developed sheath. The sheath is resultant of the retention of the egg membrane after the microfilariae extrusion from the female [55]. The sheath is important as it is protects the microfilaria body surface, therefore being related to the immune response evasion [56]. Some microfilariae of the sheated grupo, such as *Brugia*, *Wuchereria*, and *Loa* present a cephalic hook. Besides its importance for taxonomy, as this structure has an important role in the tissue penetration during microfilariariae migration and maturation in the intermediate host [57,58,59]. The putative species Onchocercidae gen. sp. 1 does not present this structure, but it is seen in Onchocercidae gen. sp. 2. The phylogenetic positioning of both putative species suggests them are close, yet different, to *Brugia* spp. and *Wuchereria bancrofti*, and the presence of the cephalic hook in Onchocercidae gen. sp. 2 microfilariae, along with other morphologic characteristics, suggest it could be included in this genus, as it was proposed in previous work [16].

Both Onchocercidae gen sp. 1 and Onchocercidae gen. sp. 2 formed a clade grouped along *Wuchereria bancrofti*, *Brugia malayi*, *Brugia pahangi*, and *Brugia timori*. Wuchereria bancrofti was endemic in Brazil, but now its transmission is limited to a few endemic areas in the metropolitan area of Recife, Pernambuco State, Northeastern Brazil, while *Brugia* spp. do not affect humans in South America [60]. According to the morphology and phylogenetic relationships of these filarids, our findings leads us to suggest that these undetermined species also inhabit the lymphatic system of their hosts. Even though there are no human cases that could be related to these species, the zoonotic potential should be considered, as several other zoonotic species with no clear relation to human health emerged in response to close interface between animals and humans [45]. The diverse human filariases origins demonstrate the plasticity of these nematodes. Their animal reservoirs are diverse: carnivores for heartworm [61]; ungulates such as cattle, horses, and wild boars for onchocercoses [62–63], and African monkeys for *Mansonella rodhaini* [64] and *Meningonema perruzzi* [65]. In forest areas, animal reservoirs are not fully identified, and the occurrence of other zoonotic species is unpredictable [46]. In the last 50 years, many species of filarial nematodes have emerged in several parts of the world as human pathogens [11,66–68], with an increasing number of cases occurring in non-endemic areas [69,70]. Future studies regarding this subject should focus on obtaining the adult parasites of the four putative species diagnosed in this study, aiming to describe them and shed light on their role to animal and human health.

The presence of *Dirofilaria immitis* in the ring-tailed coatis, an important emerging zoonositic parasite with known worldwide distribution [71], reflects another important aspect regarding this pathogen: the exchange of *D*. *immitis* between populations of domestic dogs and wild carnivores. *Dirofilaria immitis* is a common dog parasite, although it has occasionally been found parasitizing other hosts [10]. Wild carnivores are susceptible to it and generally the infections in these animals are a consequence of its presence on domestic dog populations in overlapping territories [72]. However, in the presence of competent vectors, infected wild carnivores canact as reservoir hosts [73]. In this research, the studied dogs were not infected by *D*. *immitis* and the circulation of this nematode in wild carnivores of PARNA Iguaçu may be an outcome of parasite exchange between these two hosts occurred in the past [16].

The domestic dogs infected with *Acanthocheilonema reconditum*, the only species found in these animals, were concentrated in the border area associated to the Northern region of the PARNA Iguaçu. This region is the largest inland area of the Park, with average altitude of 750m and the landscape changing from Mixed Ombrophylous Forest to Seasonal Semideciduous Forest [74,75]. The Brazilian pines *Araucaria angustifolia* are the predominant tree species in the valleys of the largest rivers, maintaining lower temperatures and higher environmental humidity for long periods along the day in comparison to the Southern areas of the Park [27].

Most filariases have dipterans as vectors, and then the prevalence of these diseases is assumed to be higher in lower altitudes, where warmer temperatures favor both the mosquito survival and the parasite development [76]. Nonetheless, the intermediate hosts of *Acanthocheilonema reconditum* are ticks, fleas, and lice instead of dipterans [77]. In the PARNA Iguaçu, six species of ticks were identified: *Amblyomma brasiliense*, *Amblyomma coelebs*, *Amblyomma incisum*, *Amblyomma ovale*, *Haemaphysalys juxtakochi*, and *Ixodes aragaoi*. For this tick community, a temporal pattern was observed, with a possible annual life cycle for most identified species, resulting in constant presence of ticks in the area, regardless of the season [78].

The results represent new host and locality records for *Acanthocheilonema reconditum* and presents four putative undetermined species that could represent new taxa of an important, yet still scarcely know group of parasites.
